# Association of assisted reproductive technology with adverse maternal outcome: A cohort study

**DOI:** 10.12669/pjms.41.1.10845

**Published:** 2025-01

**Authors:** Zhaodong Liu, Lu Yu, Xiaomeng Kang, Yulong Zhang, Jianying Yan

**Affiliations:** 1 Zhaodong Liu Department of Obstetrics and Gynecology, Fujian Maternity and Child Health Hospital College of Clinical Medicine, for Obstetrics & Gynecology and Pediatrics, Fujian Medical University, Fuzhou, Fujian Province 350001, P.R. China; 2 Lu Yu Department of Gynecologic Oncology, Fudan University Shanghai Cancer Center Xiamen Hospital, Xiamen, Fujian Province 361027, P.R. China; 3 Xiaomeng Kang Competition Activity Center of Gansu Science and Technology Museum, Lanzhou, Gansu Province 730070, P.R. China; 4 Yulong Zhang Department of Obstetrics and Gynecology, Fujian Maternity and Child Health Hospital College of Clinical Medicine, for Obstetrics & Gynecology and Pediatrics, Fujian Medical University, Fuzhou, Fujian Province 350001, P.R. China; 5 Jianying Yan Department of Obstetrics and Gynecology, Fujian Maternity and Child Health Hospital College of Clinical Medicine, for Obstetrics & Gynecology and Pediatrics, Fujian Medical University, Fuzhou, Fujian Province 350001, P.R. China

**Keywords:** Assisted reproductive Technologies, Postpartum hemorrhage, Pregnancy outcomes, Retrospective cohort study

## Abstract

**Background & Objective:**

To assess the association of assisted reproductive technologies (ART) conception with postpartum hemorrhage (PPH) during the peripartum and postpartum periods.

**Methods:**

Clinical records of 11,497 patients enrolled in Fujian Maternity and Child Health Hospital between March 2013 and December 2018 were retrospectively analyzed and divided into the ART group and the natural conception group based on the mode of conception. The incidence of PPH and blood loss at 30, 60, 90, and 120 minutes after delivery were compared.

**Results:**

Clinical records of 2527 patients with ART-derived and 78,970 patients with spontaneous pregnancies, who delivered at Fujian Maternity and Child Health Hospital between March 2013 and December 2018, were included. The incidence of PPH (2.53%vs 1.58%, P < 0.001) was greater in the ART group than in the natural conception group. ART was associated with significantly higher rates of PPH both in women who delivered vaginally and by cesarean section (*P* < 0.001). Similarly, blood loss during the delivery stage was greater in the ART group than in the naturally conceived group, regardless of the mode of delivery (P<0.001). Among women who delivered vaginally, blood loss was greater in the ART group at 30 minutes and 60 minutes after the delivery. However, blood loss at 90 and 120 minutes after the delivery was comparable in all groups, regardless of the mode of delivery.

**Conclusion:**

The use of ART was associated with poorer maternal outcomes, including increased incidence of PPH and higher blood loss at 30 and 60 minutes after the delivery.

## INTRODUCTION

Currently, over ten million children worldwide have been born with the help of assisted reproductive technologies (ART).^1^ Yet ART is associated with certain complications, including postpartum hemorrhage (PPH), gestational hypertension, preeclampsia, preterm premature rupture of membranes (PROM), gestational diabetes mellitus (GDM), intrahepatic cholestasis of pregnancy (ICP), and placenta previa, that can affect maternal and neonatal health outcomes.^2^ The mechanisms behind these associations remain complex and require further investigation to improve risk prediction and clinical management for women undergoing ART.

PPH is a particularly concerning complication of childbirth that poses significant risks to maternal health, contributing to substantial morbidity and mortality.^3^–^5^ In the United States, the incidence of PPH has been rising, largely due to an increase in cases attributed to uterine atony.^6^,^7^ Many cases of PPH requiring transfusion occur without identifiable risk factors, making maintaining a high level of clinical vigilance in all deliveries essential. A possible link between ART and the incidence of PPH underscores the importance of understanding the mechanism of this association and developing more effective preventive measures. Additionally, recent studies have shown that cesarean deliveries, especially intrapartum ones, are associated with an increased risk of developing PPH.^8^,^9^ ART is linked to a higher incidence of multiple gestations compared to natural conception^10^ and, therefore, a higher incidence of cesarean sections, further increasing the risk of PPH in this population.^11^,^12^ Consequently, assessing the impact of ART on PPH among women who deliver vaginally may help disentangle confounding variables such as multiple births and cesarean deliveries, offering clearer insights into the impact of ART on the PPH risk.

This study aimed to evaluate the association between ART and adverse maternal outcomes, with a special focus on PPH. The results of the study may contribute to guiding clinical decisions and improving patient counseling.

## METHODS

Clinical records of women who underwent ART at Fujian Maternity and Child Health Hospital between March 2013 and December 2018 were retrospectively selected from the Fujian Maternal and Child Health Hospital’s Electronic Medical Record (EMR) system. This system dates back to 2013 and provides a comprehensive record of a patient’s clinical information, including detailed data on medical history, diagnosis, treatment plan, and labor and delivery (see Supplementary file). Records of women who conceived spontaneously were used as a comparator group. Patients in both groups were further retrospectively divided based on the mode of delivery into the vaginal delivery and cesarean section subgroups.

### Ethical Approval:

The Institutional Ethics Committee approved the study, and the rights of the participants were protected (No. 2024KY201, Date: August 15, 2024).

A standardized data collection form (Proforma) was designed to ensure consistency and scientific validity of the data extracted from patients’ medical records (see Supplementary file). The form included the following sections:

- Basic patient information, such as mother’s age (years), gravidity, parity, gestational age (weeks);_ Mode of pregnancy (ART or natural conception);
- Delivery records, including mode of delivery (vaginal or cesarean), duration of delivery, details of the operation, single or multiple pregnancy, birth weight, and placental weight, etc.;
- Blood loss record at 30, 60, 90 and 120 minutes after delivery;
- Record of complications with the main focus on the occurrence of postpartum hemorrhage (PPH) and other related maternal outcomes. This data collection form was developed based on the content of the clinical record and reviewed and tested by a team of experts to ensure the accuracy and comprehensiveness of data extraction.

### Inclusion criteria:

- Women who underwent ART because of tubal factors, ovulation failure, immune infertility, genital malformation, male factors, or unexplained infertility.

### Exclusion criteria:

- Missing information (gestational age, gravidity, mode of delivery, placental weight, blood loss).
- Pregnancies that were achieved through donor oocytes.
- Pregnancies with preimplantation genetic diagnoses. The flowchart of the study design and the selection process is summarized in Fig.1.

### Outcomes of interest:

PPH, defined as blood loss with 24 hours delivery of ≥ 500 ml following vaginal delivery and ≥ 1000 ml following cesarean section,^13^ and blood loss at 30 minutes, 60 minutes, 90 minutes, and 120 minutes after the delivery. Blood loss was considered severe if it exceeded 1000 ml and very severe if it was≥2500 ml.^13^ Other complications included hypertensive disorders of pregnancy (gestational hypertension, preeclampsia, chronic hypertension complicating pregnancy, and preeclampsia superimposed upon chronic hypertension), Hemolysis, Elevated Liver enzymes and Low Platelets (HELLP) syndrome, PROM, GDM, ICP, and placenta previa.

### Statistical analysis:

SPSS 19.0 (SPSS Inc., Chicago, IL) was used for all statistical analyses. Continuous data were presented as means ±standard deviation (SD) and were compared using the independent *t-tests* or Kruskal–Wallis tests, as appropriate. Categorical variables were presented as the number of women (percentage) and were compared between the two groups using χ^2^ tests or Fisher’s exact tests, as appropriate. The results were considered statistically significant at *P*< 0.05.

## RESULTS

A total of 2527 ART-derived and 78,970 naturally conceived pregnancies delivered between March 2013 and December 2018 were identified and included in the study (Fig.1). The baseline characteristics of all groups are shown in Table-I. The ART group included 887 women who delivered vaginally and 1640 women who delivered via cesarean section. Among women in the natural conception group, 52,078 delivered vaginally, and 26,892 delivered via cesarean section. Mean maternal age, mean gestational age, gravidity, parity, and mode of delivery were significantly different between the ART and the natural conception groups (Table-I).

**Table-I:** Baseline characteristics

Baseline characteristics	ART n=2527 (%)	NC n=78790 (%)	P value
Maternal age (years, average ±S/D)	32.57±4.08	29.94±4.49	P < 0.001
Gestational age (weeks, average ±S/D)	37.07±3.63	38.29±2.98	P < 0.001
***Gravidity***			
=1	1293/2527 (51.20)	31638/78970 (40.06)	P < 0.001
>1	1231/2527 (48.71)	47274/78970 (59.86)	P < 0.001
***Parity***			
=0	2068/2527 (81.84)	43748/78970 (55.40)	P < 0.001
>1	377/2527 (14.91)	35222/78970 (44.60)	P < 0.001
***Mode of delivery***			
Instrumental vaginal	38/2527 (1.50)	1195/78970 (1.51)	P=0.969
Cesarean section	1640/2527 (65.00)	26891/78970 (34.05)	P < 0.001
Vaginal delivery	846/2527 (33.50)	50841/78970 (64.38)	P < 0.001
Regional analgesia	14/2527 (0.55)	479/78970 (0.61)	
Birth weight (g)	2903.95	3165.70	
<3000	1253/2527 (49.58)	21882/78970 (27.71)	P < 0.001
3000-3499	798/2527 (31.58)	34763/78970 (44.02)	P < 0.001
3500-3999	439/2527 (17.37)	19150/78970 (24.25)	P < 0.001
≥4000	94/2527 (3.71)	221/78970 (0.28)	P < 0.001
Placental weight (g, average ±S/D)	736.67±241.86	628.24±3169.18	P < 0.001
***Gestational complications***			
Hypertensive disorders of pregnancy	218/2527 (8.62)	2886/78970 (3.65)	P < 0.001
Gestational hypertension	72/2527 (2.85)	1255/78970 (1.60)	P < 0.001
Preeclampsia	127/2527 (4.95)	1355/78970 (1.71)	P < 0.001
Chronic hypertension complicating pregnancy	12/2527 (0.47)	183/78970 (0.23)	P=0.014
Preeclampsia superimposed upon chronic hypertension	7/2527 (0.30)	90/78970 (0.11)	P=0.019
HELLP syndrome	5/2527 (0.20)	69/78970 (0.09)	P=0.069
Premature rupture of membranes	622/2527 (24.61)	19286/78970 (24.42)	P=0.825

The ART group also included higher proportions of nulliparous women and women who delivered via cesarean section. As shown in Table-I, the incidences of adverse maternal outcomes, including gestational hypertension, preeclampsia, PPH, GDM, ICP, and placenta previa, were considerably greater in the ART group than in the natural conception group (*P<0.05* for all). However, ART was not associated with an increased risk of PROM and HELLP syndrome (*P* > 0.05). The frequency of PPH was also analyzed according to the mode of delivery. ART was associated with a significantly higher rate of PPH both in women who delivered vaginally and via cesarean section compared to natural conception (*P* < 0.001).

There was a significantly higher incidence of blood loss of 500–1000 ml in the vaginal delivery group (Table-II). In contrast, more women reported blood loss of 1000–1500 ml in the cesarean section group (Table-III) compared with other categories of blood loss. Blood loss at different delivery stages in women who delivered vaginally or via cesarean section is shown in Table-IV. Blood loss during the delivery stage was greater in the ART group than in the natural conception group for both vaginal and cesarean deliveries (*P* < 0.001). Among women who delivered vaginally, blood loss was greater in the ART group at 30 minutes, 45 minutes, and 60 minutes after the delivery (*P* < 0.004). In the cesarean section subgroup, blood loss was greater in the ART group than in women who conceived naturally at 60 minutes after the delivery (*P*=0.005), while the blood loss at 90 and 120 minutes after the delivery was comparable regardless of the mode of delivery (Table-V).

**Table-II:** Amount of bleeding: vaginal delivery.

Amount of bleeding (ml)	No (%) of PPH		P value

	ART (Vaginal delivery) n = 887 (%)	NC (Vaginal delivery) n=52078 (%)	
500-	52/887(5.86)	1019/52078(1.96)	P<0.001
1000-	6/887(0.67)	152/52078(0.29)	P=0.037
1500-	5/887(0.56)	45/52078(0.09)	P<0.001
2000-	1/887(0.11)	17/52078(0.03)	P=0.199
2500-	1/887(0.11)	14/52078(0.03)	P=0.172
Total	64/887(7.21)	1247/52078(2.39)	P <0.001

***Note:*** PPH Postpartum hemorrhage, ART the assisted reproductive technologies group, NC the natural conception group.

**Table-III:** Amount of bleeding in Cesarean section.

Amount of bleeding (ml)	No (%) of PPH		P value

	ART (Cesarean section) n=1640 (%)	NC (Cesarean section) n=26892 (%)	
1000-	22/1640(1.34)	144/26892(0.53)	P=0.001
1500-	8/1640(0.37)	45/26892(0.17)	P=0.020
2000-	1/1640(0.06)	49/26892(0.18)	P=0.254
2500-	1/1640(0.06)	22/26892(0.09	P=0.46
3000-	0/1640	15/26892(0.06)	P=0.339
Total	32/1640(1.95)	260/26892(0.97)	P<0.001

***Note:*** PPH Postpartum hemorrhage, ART the assisted reproductive technologies group, NC the natural conception group.

**Table-IV:** Blood loss at different stages of vaginal and Caesarean delivery.

Stage	Blood loss at different stages of vaginal delivery.			Blood loss at different stages of Cesarean delivery.		

	ART (Vaginal delivery) n=887 (ml, average ±S/D)	NC (Vaginal delivery) n=52078 (ml, average ±S/D)	P value	ART (Caesarean section) n=1640(ml, average ±5/D)	NC (Caesarean section) n=26892(ml, average ±5/D)	P value
delivery	142.7±125.20	106.65±70.81	P<0.001	449.23±204.18	401.96±229.19	P<0.001
30 minutes after delivery	59.49±93.17	39.64±52.58	P<0.001			
45 min after delivery	23.37±36.86	18.41±22.80	P<0.001			
60 min after delivery	14.73±25.98	12.23±20.20	P=0.004	13.41±49.53	9.96±34.96	P=0.005
90 min after delivery	10.56±47.83	8.54±23.46	P=0.213	9.84±21.54	8.80±25.39	P=0.060
120 min after delivery	6.78±15.34	6.58±17.63	P=0.702	9.57±24.81	8.89±16.51	P=0.270
Total	257.64±219.85	192.06±132.07	P<0.001	482.05±216.84	426.61±240.06	P<0.001

***Note:*** ART the assisted reproductive technologies group, NC the natural conception group.

**Table-V:** The incidence of PPH after vaginal and Caesarean delivery.

Gestational age (weeks)	The incidence of PPH after vaginal delivery			The incidence of PPH after Caesarean delivery		

	No (%) of PPH			No (%) of PPH		

	ART (Vagina l delivery) n=887 (%)	NC (Vaginal delivery) n=52078 (%)	P value	IVF (Caesarean delivery) n=1640 (%)	NC (Caesarean delivery) n=26892 (%)	P value
34-35	1/39 (2.56)	17/1227 (1.39)	P=0.540	5/149 (3.36)	22/1146 (1.92)	P <0.001
36-37	8/87 (9.20)	82/3929 (2.09)	P<0.001	11/502 (2.19)	79/3708 (2.13)	P=0.929
38-39	21/354 (5.93)	513/24200 (2.12)	p<0.001	8/680 (1.18)	93/14904 (0.62)	P <0.001
>40	22/238 (9.24)	544/19421 (2.80)	P<0.001	4/219 (1.83)	46/6234 (0.74)	P <0.001
Tota l	64/887 (7.22)	1247/52078 (2.39)	P<0.001	32/1640 (1.95)	260/26892 (0.97)	P <0.001

***Note:*** PPH Postpartum hemorrhage, ART the assisted reproductive technologies group, NC the natural conception group.

## DISCUSSION

This study revealed a significant association between assisted reproductive technology (ART) and a higher incidence of gestational complications. Pregnancies conceived via ART were linked to an elevated risk of postpartum hemorrhage (PPH), regardless of delivery mode (vaginal or cesarean). Additionally, the frequencies of all gestational hypertension disorders were higher in women who conceived through ART.

The findings of this study support previous reports that have shown a connection between ART and an increased incidence of gestational hypertension and preeclampsia.^14^–^16^ Johnson et al. reported reduced vascular density in the maternal and fetal placenta in ART pregnancies,^17^ suggesting that impaired placental vascularization associated with ART may contribute to the observed increase in gestational hypertension.

**Supplementary Fig.1:**
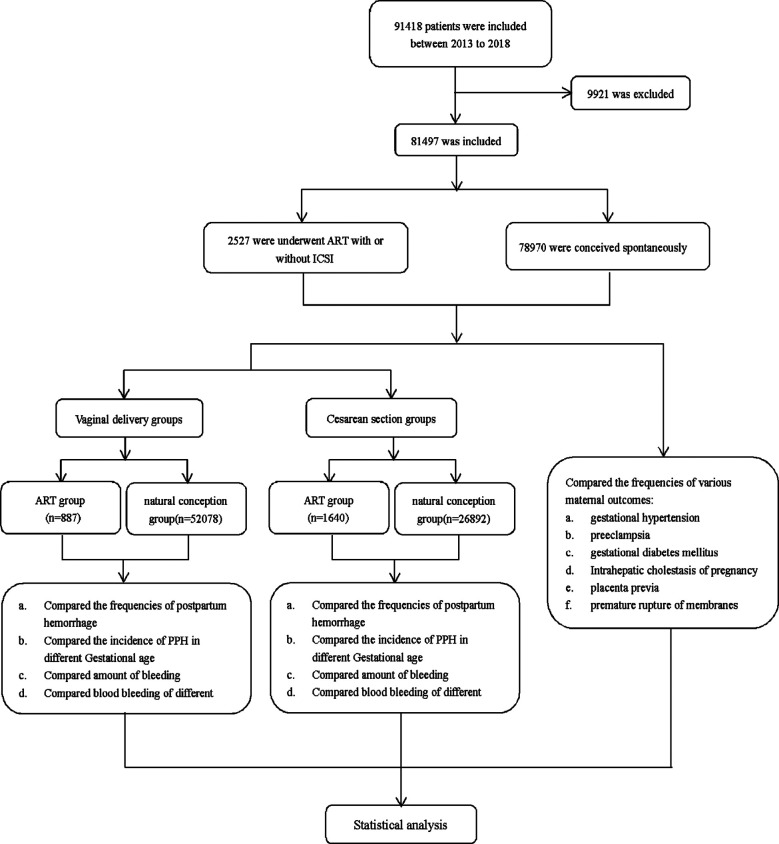
The flowchart of the study design and the selection process.

This study also indicates that women who conceive through ART are more likely to develop GDM compared with those who conceive naturally, which is consistent with earlier research.^18^,^19^ Advanced maternal age and multiple pregnancies are established risk factors for GDM and adverse perinatal outcomes.^20^–^22^ In this cohort, women in the ART group tended to be older than those in the natural conception group, which may partially explain these findings, as age-related factors like increased insulin resistance, oxidative stress, and inflammatory markers contribute to GDM development. Further studies are warranted to investigate the association between ART and GDM.

The incidence of ICP, a common but typically self-resolving liver condition during pregnancy, was notably higher in the ART group, similar to previous studies.^23^ Research suggests that serum bile acid levels in women with ICP may be higher in ART pregnancies compared to spontaneous pregnancies,^24^ indicating that a greater incidence of ICP may predispose ART pregnancies to other adverse outcomes. However, due to the low number of ICP cases in this study, additional research with larger samples is needed to explore this potential association further.

The study also observed a higher rate of placenta previa in ART pregnancies. While some studies have suggested that the mechanical embryo transfer involved in ART could increase the risk of lower uterine implantation and placenta previa,^25^,^26^ others found no such association.^27^ The inconsistencies may stem from the relatively small sample sizes of these studies. In contrast, this research provided data on 2,527 ART pregnancies and 78,790 natural conceptions, which enhances its reliability. Nonetheless, further high-quality studies are needed to clarify the link between ART and placenta previa.

The relationship between ART and PPH remains inconclusive, with several studies finding no association, contradicting the observations in this study.^19^,^28^ The lack of association in some previous studies may result from their smaller sample sizes. This study, however, included a significantly larger cohort and showed that ART was associated with PPH regardless of the delivery mode, consistent with findings from other studies.^29^,^30^ It is plausible that ART itself or factors related to infertility, such as submucosal or large intramural myomas and adenomyosis—conditions that can impair uterine function—may increase the risk of PPH.23

Additionally, this study demonstrated that women in the ART group who delivered vaginally and experienced PPH were older than those in the natural conception group (32.57 ± 4.08 vs. 29.94 ± 4.49 years), in line with previous research.^14^,^19^ Since uterine contractility tends to decrease with age, this may contribute to the higher frequency of PPH in vaginal deliveries among the ART group. Consistent with other reports,^23^,^31^ ART pregnancies were also more likely to involve nulliparous women, which could partly explain the increased rate of PPH.

The large sample size and the clear diagnostic definitions of PPH and blood loss are notable strengths of this study. A subgroup analysis based on delivery mode provided valuable insights into the impact of ART on PPH frequency among women delivering vaginally, offering clinical implications for delivery mode selection and risk assessment in ART pregnancies. Additionally, identifying 60 minutes post-delivery as the time with the most blood loss could aid clinicians in monitoring and preventing PPH in this group.

### Limitations:

Only women who underwent ART or delivered at the study hospital were included, which may limit the generalizability of the findings. Additionally, different ART procedures—such as IVF, intracytoplasmic sperm injection, and pre-implantation genetic diagnosis—were not distinguished. Lastly, other factors, such as the prevalence of polycystic ovary syndrome within the cohort, which could contribute to adverse pregnancy outcomes, were not accounted for. Future studies should explore additional risk factors in ART pregnancies and their potential mechanisms.

## CONCLUSION

This study found a significant association between ART and several adverse maternal outcomes, such as gestational hypertension, preeclampsia, GDM, ICP, placenta previa, and PPH. ART was associated with a higher incidence rate of PPH regardless of the delivery mode. Finally, the study found that most blood loss occurred within 60 minute after the delivery. Further studies are needed to examine the mechanisms underlying the association between ART and adverse maternal outcomes, including PPH.

### Authors’ contributions:

**ZL** and **LY:** Design of study. Literature search and prepared the article. **XK**, **YZ** and **JY:** Collected the data. Performed data interpretation and analysis, critical review. **JY:** Critical review. preparation of the manuscript. All authors have read the final version and are responsible and accountable for the accuracy and integrity of the work.
